# Redundancy of myostatin and growth/differentiation factor 11 function

**DOI:** 10.1186/1471-213X-9-24

**Published:** 2009-03-19

**Authors:** Alexandra C McPherron, Thanh V Huynh, Se-Jin Lee

**Affiliations:** 1 Genetics of Development and Disease Branch, National Institute of Diabetes and Digestive and Kidney Diseases, National Institutes of Health, 9000 Rockville Pike, Bethesda, MD 20892, USA; 2 Department of Molecular Biology and Genetics, Johns Hopkins University School of Medicine, 725 North Wolfe Street, Baltimore, MD 21205, USA

## Abstract

**Background:**

Myostatin (Mstn) and growth/differentiation factor 11 (Gdf11) are highly related transforming growth factor β (TGFβ) family members that play important roles in regulating embryonic development and adult tissue homeostasis. Despite their high degree of sequence identity, targeted mutations in these genes result in non-overlapping phenotypes affecting distinct biological processes. Loss of *Mstn *in mice causes a doubling of skeletal muscle mass while loss of *Gdf11 *in mice causes dramatic anterior homeotic transformations of the axial skeleton, kidney agenesis, and an increase in progenitor cell number in several tissues. In order to investigate the possible functional redundancy of myostatin and Gdf11, we analyzed the effect of eliminating the functions of both of these signaling molecules.

**Results:**

We show that *Mstn*^-/- ^*Gdf11*^-/- ^mice have more extensive homeotic transformations of the axial skeleton than *Gdf11*^-/- ^mice in addition to skeletal defects not seen in single mutants such as extra forelimbs. We also show that deletion of *Gdf11 *specifically in skeletal muscle in either *Mstn*^+/+ ^or *Mstn*^-/- ^mice does not affect muscle size, fiber number, or fiber type.

**Conclusion:**

These results provide evidence that myostatin and Gdf11 have redundant functions in regulating skeletal patterning in mice but most likely not in regulating muscle size.

## Background

Myostatin (Mstn) and growth/differentiation factor 11 (Gdf11) are highly related members of the transforming growth factor β (TGFβ) superfamily of secreted growth and differentiation factors. Like other TGFβ family members, myostatin and Gdf11 precursor proteins are proteolytically processed to form biologically-active carboxy-terminal dimers. Myostatin and Gdf11 share 90% amino acid identity in this carboxy-terminal region which places them in their own TGFβ family subgroup. They also have similar signaling pathways; both bind the activin type IIB receptor (Acvr2b, also known as ActRIIB) and activate the intracellular mediator Smad 2/3 pathway [1-5], and both are antagonized by follistatin, a secreted glycoprotein that can bind several TGFβ family members [2,6-9].

Mstn is predominantly expressed in developing and adult skeletal muscle [10]. We previously reported the disruption of the *Mstn *gene by gene targeting in mice and showed that myostatin normally functions as a negative regulator of skeletal muscle mass [10]. Most *Mstn*^-/- ^muscles are approximately double the mass of *Mstn*^+/+ ^muscles due to both hyperplasia and hypertrophy of myofibers [10]. Subsequently, naturally occurring *Mstn *gene mutations were found in cattle, dogs, sheep, and one child with increased muscle mass indicating conservation of myostatin function in mammals [11]. Injection of myostatin antagonists also causes a significant increase in muscle mass in normal and dystrophic adult mice demonstrating that myostatin function is not restricted to developmental stages [12-17]. These results suggest that inhibition of myostatin may be a promising therapeutic target for treating muscle wasting diseases.

We also reported the deletion of the *Gdf11 *gene by gene targeting in mice [18]. In contrast to *Mstn*^-/- ^mice, *Gdf11*^-/- ^mice display anterior homeotic transformations of the axial skeleton wherein the identity of posterior vertebrae are transformed into those of more anterior vertebrae [18]. Anterior/posterior (A/P) axial mesoderm identity is thought to be established by differential combinatorial expression of *Hox *genes that are induced as cells move through the primitive streak during gastrulation [19]. Consistent with this model, *Gdf11 *is strongly expressed in the primitive streak and tail bud [7,18,20], and *Gdf11*^-/- ^mice have altered *Hox *gene expression along the A/P axis [18]. In addition to the axial skeleton, defects in *Gdf11*^-/- ^mice are found in other tissues where *Gdf11 *is expressed. For instance, *Gdf11*^-/- ^mice have renal agenesis, an increase in the number of islet progenitors in the pancreas, an increase in the number of neurons and neuronal progenitors in the olfactory epithelium, and an increase in the number of retinal ganglion cells and reduction in the number of photoreceptors and amacrine cells in the retina [21-25].

Although the phenotypes caused by each gene disruption appear to be non-overlapping, the high sequence identity and similarity in signaling mechanisms suggest the two factors may be functionally redundant. In addition, *Mstn *is expressed transiently in the primitive streak in the chick [26]. We therefore decided to test whether myostatin has a role in determining A/P positional identity of the axial skeleton. In a similar vein, Gdf11 has been shown to inhibit myogenesis in chick limb mensenchyme cultures [27], so we also sought to test whether Gdf11 has a role in regulating skeletal muscle mass. Our data show that myostatin and Gdf11 have redundant functions in regulating skeletal patterning in mice but most likely not in regulating muscle size.

## Results and discussion

### Skeletal patterning in *Mstn*^-/- ^*Gdf11*^-/- ^mice

**Figure 1:**
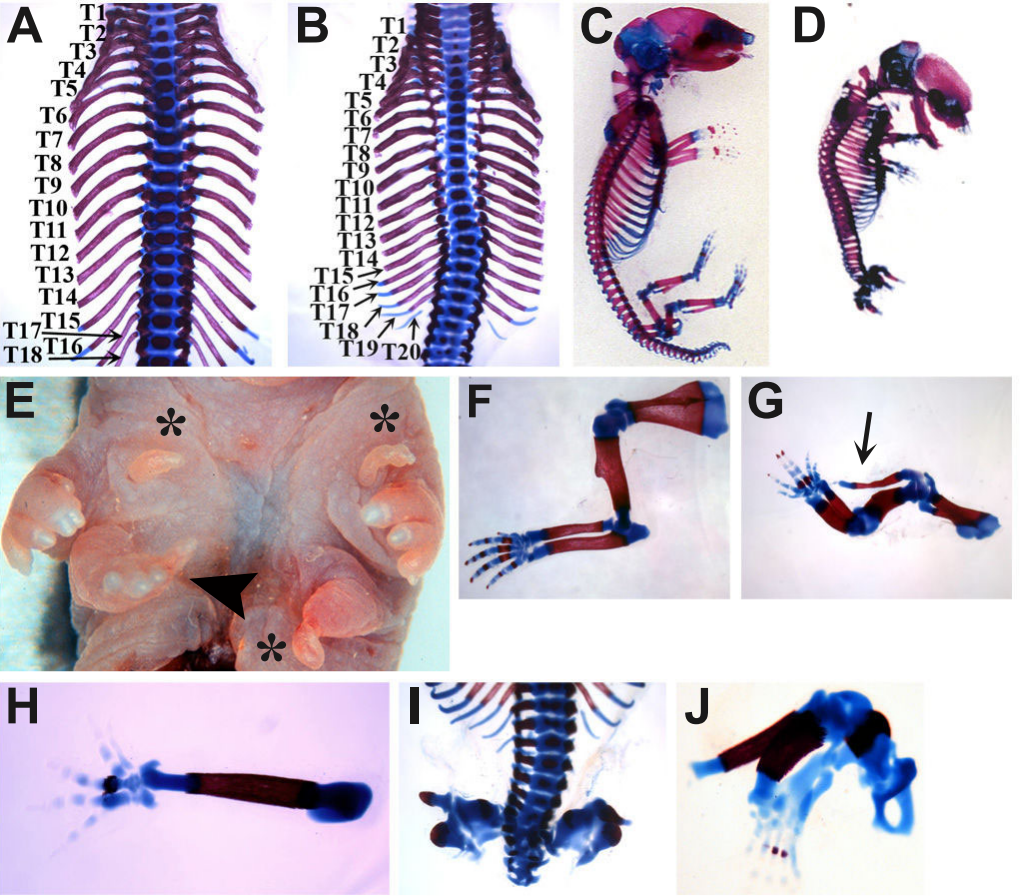
**Skeletal defects in *Mstn*^-/- ^*Gdf11*^-/- ^newborn mice**. Thoracic region of *Gdf11*^-/- ^(A) and *Mstn*^-/- ^*Gdf11*^-/- ^(B) mutants showing the increase in rib number. Note that the anterior homeotic transformations of thoracic vertebrae are more extensive in the *Mstn*^-/- ^*Gdf11*^-/- ^mutant (20 ribs) versus the single *Gdf11*^-/-^mutant (18 ribs). Whole skeleton preparation of *Gdf11*^-/- ^(C) and *Mstn*^-/- ^*Gdf11*^-/- ^(D) pups. (E) Ventral torso of newborn double mutant showing multiple projections from the skin (*) and an extra limb (arrowhead). Forelimb skeleton preparations of *Gdf11*^-/- ^(F) and *Mstn*^-/- ^*Gdf11*^-/- ^(G and H) pups showing an extra bone emanating from the shoulder of the double mutant (G, arrow) and an extra limb (H). The limb in (G) has been rotated for a better view of the extra bone. (I and J) Hindlimb phenotypes of *Mstn*^-/- ^*Gdf11*^-/- ^pups. Note the truncation of the vertebral column (I) and malformation of the ilieum and all leg bones (J).

We crossed double heterozygous mice (*Mstn*^+/- ^*Gdf11*^+/-^) to produce *Mstn*^-/- ^*Gdf11*^-/- ^mice to examine redundancy of myostatin and Gdf11 function during development. *Mstn*^-/- ^*Gdf11*^-/- ^mice were born at the expected ratio (data not shown), but none were found alive. While most *Gdf11*^-/- ^mutants have renal agenesis and cleft palate [22], these phenotypes were fully penetrant in *Mstn*^-/- ^*Gdf11*^-/- ^mice. To analyze skeletal alterations in double mutants, we performed skeleton preps on newborn mice to determine vertebral identity. *Wild-type *mice commonly have 13 thoracic and 6 lumbar vertebrae while *Gdf11*^-/- ^mice typically have 18 thoracic and 8 lumbar vertebrae [18]. *Gdf11*^-/- ^offspring from crosses of double heterozygotes had 17–19 thoracic and 7–9 lumbar vertebrae as expected (Tables 1 and 2, Figure 1A). In contrast, skeletons of *Mstn*^-/- ^mice had a similar pattern as the majority of *wild-type *mice with 13 thoracic and 6 lumbar vertebrae (Tables 1 and 2). Deletion of one allele of *Mstn *in *Gdf11*^-/- ^mice (*Mstn*^+/- ^*Gdf11*^-/-^) also had no effect on thoracic or lumbar vertebral number in comparison with *Gdf11*^-/- ^mice (Tables 1 and 2). Loss of both alleles of *Mstn *in *Gdf11*^-/- ^mice, however, resulted in a much more severe phenotype. Most *Mstn*^-/- ^*Gdf11*^-/- ^mice had 20 rather than 18 thoracic vertebrae although in 4 out of 17 double mutant pups the transformations were even more dramatic (Figure 1B, Table 1). In these 4 mice, the most posterior thoracic vertebra was asymmetric with a 21^st ^rib on one side and a lumbar-like phenotype on the other (Table 1 and 2). The extent of the anterior homeotic transformations in the thoracic region of *Mstn*^-/- ^*Gdf11*^-/- ^mice were greater than any we have seen in *Gdf11*^-/- ^mice.

**Table 1:** Thoracic vertebral number from newborn mice

	Genotype								
	
*Mstn*	+/+	+/+	+/+	+/-	+/-	+/-	-/-	-/-	-/-
*Gdf11*	+/+	+/-	-/-	+/+	+/-	-/-	+/+	+/-	-/-
Total number of thoracic vertebrae	Number of animals								

13	10	-	-	10	-	-	10	1	-
14	-	10	-	-	10	-	-	9	-
15	-	-	-	-	-	-	-	-	-
16	-	-	-	-	-	-	-	-	-
17	-	-	-	-	-	-	-	-	-
17+18	-	-	1	-	-	-	-	-	-
18	-	-	8	-	-	7	-	-	-
18+19	-	-	-	-	-	1	-	-	-
19	-	-	1	-	-	2	-	-	1
19+20	-	-	-	-	-	-	-	-	1
20	-	-	-	-	-	-	-	-	11
20+21	-	-	-	-	-	-	-	-	4

Numbers of mice displaying a given number of vertebrae are shown. Some mice have left-right asymmetry of the most posterior thoracic vertebra with a rib on one side and a lumbar phenotype on the other. These are designated by a + sign between 2 numbers indicating the number of ribs for each side.

**Table 2:** Lumbar vertebral number from newborn mice

	Genotype								
	
*Mstn*	+/+	+/+	+/+	+/-	+/-	+/-	-/-	-/-	-/-
*Gdf11*	+/+	+/-	-/-	+/+	+/-	-/-	+/+	+/-	-/-
Total number of lumbar vertebrae	Number of animals								

5	3	-	-	3	-	-	-	-	
5+6	1	-	-	-	-	-	-	-	
6	6	10	-	7	10	-	10	9	
6+7	-	-	-	-	-	-	-	-	
7	-	-	1	-	-	1	-	1	
7+8	-	-	1	-	-	-	-	-	
8	-	-	5	-	-	5	-	-	
8+9	-	-	2	-	-	-	-	-	
9	-	-	1	-	-	3	-	-	
9+10	-	-	-	-	-	1	-	-	

Numbers of mice displaying a given number of vertebrae are shown. Some mice have left-right asymmetry of the most posterior lumbar vertebra with a lumbar phenotype on one side and a sacral phenotype the other. These are designated by a + sign between 2 numbers indicating the number of vertebrae possessing a lumbar appearance for each side. Asymmetric vertebrae with a thoracic and lumbar phenotype were counted as thoracic (Table 1). Number of lumbar vertebrae could not be determined for *Mstn*^-/- ^*Gdf11*^-/- ^mice.

The skeletal regions posterior to the thoracic region were also altered in mutant mice. As previously described [18], the sacral vertebrae were present in *Gdf11*^-/- ^mice, but the axis was truncated in the caudal region resulting in an overall decrease in the number of vertebral segments and the loss of most of the tail with only a few deformed vertebrae remaining (Figure 1C). In *Mstn*^-/- ^*Gdf11*^-/- ^mice, there were even fewer post-thoracic vertebrae with an average of 10 post-thoracic segments, the most posterior of which were malformed (Figure 1D and 1I). The sacral vertebrae were not identifiable in *Mstn*^-/- ^*Gdf11*^-/- ^mice making it difficult to tell which vertebra was the most posterior lumbar vertebra (Figure 1I). The increase in severity of the *Gdf11 *null phenotype throughout the vertebral axis in the absence of *Mstn *demonstrates that myostatin and Gdf11 play redundant roles in patterning and development of the axial skeleton.

*Mstn*^-/- ^*Gdf11*^-/- ^pups also had other skeletal defects not seen in *Gdf11*^-/- ^mutants. The frontal bones curved anteriorly to meet the nasal bone which gave the skull vault a rounded shape compared to *Gdf11*^-/- ^mice (Figure 1C and 1D and Additional file 1A and 1B: Cranial and forelimb digit skeletal defects in *Mstn*^-/- ^*Gdf11*^-/- ^newborn mice). Although *Gdf11 *is expressed in the developing limb buds [7,20], no defects were detected in limbs of *Gdf11*^-/- ^mutants (Figure 1C and 1F)*. Mstn*^-/- ^*Gdf11*^-/- ^mice, however, had severe limb defects. Many *Mstn*^-/- ^*Gdf11*^-/- ^mice had small projections emanating from the skin on the ventral surface of the torso, some of which were filled with a single rod of cartilage (Figure 1E). In *Mstn*^-/- ^*Gdf11*^-/- ^mutants, the long bones of the forelimb were shortened relative to those in *Gdf11*^-/- ^mice and, in 9 out of 18 double mutants, an extra bone projected from shoulder (Figure 1F and 1G). The most surprising result was a third limb that resembled a forelimb composed of a single unidentifiable long bone with attached digits (Figure 1E and 1H). This phenotype was found in 6 out of 18 double mutants, two of which also had an extra bone on the contralateral shoulder. In all *Mstn*^-/- ^*Gdf11*^-/- ^mutants, the forelimbs in the normal position displayed digital patterning defects including a sixth digit, which appeared to be similar to digit V, and syndactyly of digits III and IV consistent with the expression of *Gdf11 *in the interdigital region where programmed cell death would normally occur [7,20] (Figure 1G and Additional file 1C and 1D: Cranial and forelimb digit skeletal defects in *Mstn*^-/- ^*Gdf11*^-/- ^newborn mice). No other genotypes displayed any of these limb defects.

The hindlimbs in *Mstn*^-/- ^*Gdf11*^-/- ^mice displayed very different malformations than the forelimbs. The hindlimbs were small and deformed although what appeared to be elements of both proximal and distal structures, pelvic bones, long bones, and digits, were seen in most double mutants (Figure 1D, I and 1J). These results suggest some proximal/distal patterning was maintained despite inhibition of hindlimb bud outgrowth.

This is the first demonstration that myostatin and Gdf11 are both required for limb development and axial skeletal development and patterning. The effects of deletion of both factors on the axial skeleton was greater than for loss of *Gdf11 *alone most likely due to the transient expression of *Mstn *in the posterior primitive streak overlapping with *Gdf11 *expression [26]. We presume that the extra limbs seen in the double mutant mice result from an expansion of the limb field, but clearly additional studies will be required to fully understand the molecular basis for the formation of the extra limb buds.

### Skeletal muscle in *Gdf11 *mutants

**Figure 2:**
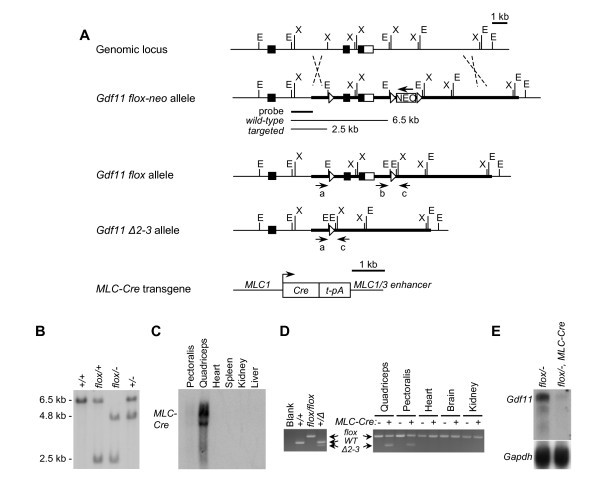
**Muscle-specific targeting of *Gdf11 *gene**. (A) Representation of targeting strategy. The 3 exons are shown as boxes with coding sequences shaded black and the 3'UTR open. The targeting construct is represented by a thick line which contains *lox*P sequences with *Eco*RI restriction sites inserted into the second *Xba*I site in exon 1 and flanking a *neo *gene inserted into the *Eco*RI site downstream of the 3'UTR. Cre-mediated recombination of the *lox*P sites flanking the *neo *gene results in a *Gdf11*^*flox *^allele. Recombination of the *Gdf11*^*flox *^allele generates the *Gdf11*^Δ2–3 ^allele. Oligonucleotide primers used for distinguishing alleles are labeled a, b, and c. A skeletal muscle-specific *Cre *expressing transgene was constructed using the *MLC1 *promoter/1/3 enhancer and an SV40 t antigen intron and poly adenylation signal. (B) Southern blot showing detection of *Gdf11*^+^, *Gdf11*^*flox*^, and *Gdf11*^-^(null, from the original knockout line) alleles. (C) Northern blot analysis of *MLC-Cre *transgene expression in pectoralis and quadriceps muscles but not in other tissues. (D) Detection of *Gdf11 *alleles in genomic DNA in *Gdf11*^*flox*/*flox*^and *Gdf11*^*flox*/*flox*^*MLC-Cre *mice by PCR (right panel). Recombination was detected in quadriceps and pectoralis muscles of *Gdf11*^*flox*/*flox*^*MLC-Cre *mice but not in heart, brain, or kidney. No recombination was seen in *Gdf11*^*flox*/*flox*^mice. Left panel shows control reactions. (E) Northern blot analysis of skeletal muscle *Gdf11 *expression showing a strong decrease in expression in *Gdf11*^*flox*/-^*MLC-Cre *muscle compared to *Gdf11*^*flox*/-^muscle.

We also sought to determine whether myostatin and Gdf11 are functionally redundant with respect to control of muscle mass. The increase in muscle mass in *Mstn*^-/- ^mice is not present at birth (A.C.M. and S-J.L., unpublished observations) so the neonatal death of *Gdf11*^-/- ^and *Mstn*^-/- ^*Gdf11*^-/- ^mice precludes a comparison of the skeletal muscle phenotype. We therefore generated a targeting construct containing a conditional deletion allele of the *Gdf11 *gene by inserting *lox*P recombination sites into intron 1 and flanking a *neo *gene downstream of the *Gdf11 *3' UTR (*Gdf11*^*flox*-*neo*^) (Figure 2A and 2B). Following homologous recombination in embryonic stem (ES) cells and injection of the targeted cells into blastocysts, we obtained chimeric mice that transmitted the *Gdf11*^*flox*-*neo *^allele through the germline. Mice carrying the *Gdf11*^*flox*-*neo *^allele were then crossed to *EIIa-Cre *transgenic mice to generate *Gdf11*^*flox*/+ ^mice carrying one upstream and one downstream *lox*P site after removal of the *neo *gene. Recombination at the remaining *lox*P sites would be predicted to delete exons 2 and 3 (*Gdf11*^Δ2–3^), which would remove the biologically-active carboxy-terminal domain. To demonstrate that recombination of *lox*P sites produced a null allele, we generated germline recombination of *lox*P sites in *Gdf11*^*flox*/+ ^mice (*Gdf11*^Δ2–3/+^). Skeletal analysis performed on offspring from *Gdf11*^Δ2–3/+ ^matings showed that *Gdf11*^Δ2–3/+ ^and *Gdf11*^Δ2–3/Δ2–3 ^mice had 1 and 5 extra thoracic vertebrae, respectively (data not shown). These thoracic vertebral numbers are identical to that of *Gdf11*^+/- ^and *Gdf11*^-/- ^mice [18] confirming that recombination of *lox*P sites in the *Gdf11*^*flox *^allele results in a null allele.

For skeletal muscle-specific recombination of *lox*P sequences, a Cre deletor transgenic line was made using the myosin light chain 1/3 promoter/enhancer (*MLC-Cre*) (Figure 2A). As expected, *Cre *expression in this line was restricted to skeletal muscle (Figure 2C). *MLC-Cre *mice were crossed to *Gdf11*^*flox*/+ ^mice and then backcrossed to *Gdf11*^*flox*/+ ^mice or crossed to *Gdf11*^+/- ^from the original knockout line. Recombination of *Gdf11*^*flox *^was detected specifically in skeletal muscle genomic DNA (Figure 2D). In *Gdf11*^*flox*/- ^muscle, Cre-mediated recombination resulted in a near complete reduction in skeletal muscle *Gdf11 *expression (Figure 2E).

**Figure 3:**
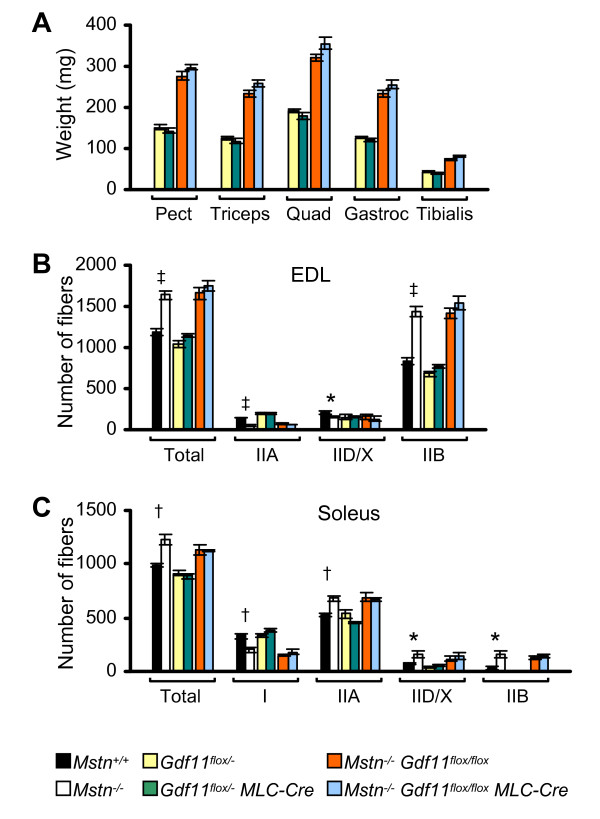
**Muscle weight, fiber number, and fiber type in skeletal muscle-specific *Gdf11 *mutant mice in a *Mstn *wild-type or null background**. (A) Weight of pectoralis, triceps, quadriceps, gastrocnemius/plantaris, and tibialis anterior muscles (*n *= 7–12). (B) Number of total, IIA, IID/X, and IIB fibers in the EDL muscle (*n *= 3–4). EDL muscles had on average less than 4 type I fibers in all genotypes so type I data are not shown. (C) Number of total, type I, IIA, IID/X, and IIB fibers in the soleus muscle (*n *= 3–5). Skeletal muscle-specific *Gdf11 *deletion had no effect on muscle mass, fiber number, or fiber type unlike *Mstn *deletion (*P < 0.05, ^†^*P *< 0.01, ^‡^*P *< 0.001). Data are mean ± s.e.

Body weight and muscle mass were measured in all 8 possible genotypes produced from crosses of *Gdf11*^*flox*/+ ^*MLC-Cre *and *Gdf11*^+/- ^mice. There were no differences in body weight or muscle mass between *Gdf11*^+/+^, *Gdf11*^+/+ ^*MLC-Cre*, *Gdf11*^+/-^, *Gdf11*^+/- ^*MLC-Cre*, *Gdf11*^*flox*/+^, or *Gdf11*^*flox*/+ ^*MLC-Cre *mice demonstrating that there was no heterozygous or transgene phenotype (data not shown). We next performed an examination of the muscle phenotype of *Gdf11*^*flox*/- ^and *Gdf11*^*flox*/- ^*MLC-Cre *mice. There was no statistically significant difference in body weight or muscle mass between *Gdf11*^*flox*/- ^and *Gdf11*^*flox*/- ^*MLC-Cre *mice (data not shown and Figure 3A).

Skeletal muscles vary in their composition of fiber types which differ in metabolic and contractile properties [28]. In mice, type I fibers are slow contracting and oxidative, type IIB fibers are fast contracting and glycolytic, and type IIA and IID/X fibers are intermediate in phenotype. *Mstn*^-/- ^mice have an overall increase in the number of muscle fibers and alterations in the proportions of the different fiber types compared to *Mstn*^+/+ ^mice resulting in a shift toward more glycolytic muscle [10,29,30]. To determine whether Gdf11 regulates fiber number or type, we counted the number of each of the four fiber types in *Gdf11*^*flox*/- ^and *Gdf11*^*flox*/- ^*MLC-Cre *mice from the widest part of each muscle. We chose a fast muscle, the extensor digitorum longus (EDL), and a slow muscle, the soleus, for analysis because they have been previously shown to have alterations in fiber number and type in *Mstn*^-/- ^mice [29,30]. Unlike muscle in *Mstn*^-/- ^mice, there was no difference in the total number of fibers in either the EDL or the soleus muscles between *Gdf11*^*flox*/- ^and *Gdf11*^*flox*/- ^*MLC-Cre *mice (Figure 3B and 3C). Fiber type composition in *Gdf11*^*flox*/- ^and *Gdf11*^*flox*/- ^*MLC-Cre *mice was not significantly different either (Figure 3B and 3C).

Because a role for myostatin in axial skeletal patterning was found only in the absence of Gdf11, we asked whether a phenotype caused by loss of Gdf11 in muscle might be present in the absence of myostatin. *Mstn*^-/- ^*Gdf11*^*flox*/*flox *^mice were crossed to *Mstn*^-/- ^*Gdf11*^*flox*/*flox *^*MLC-Cre *mice, and body weight and muscle mass were measured in offspring at 8 weeks and 6 months of age. There were no significant differences in body weight or muscle mass found between genotypes at either age (data not shown and Figure 3A). Next, we determined fiber number and fiber composition. There were no significant differences in the total number of fibers or in the number of individual fiber types in EDL or soleus between *Mstn*^-/- ^*Gdf11*^*flox*/*flox *^and *Mstn*^-/- ^*Gdf11*^*flox*/*flox *^*MLC-Cre *mice (Figure 3B and 3C).

Our results provide evidence that that myostatin and Gdf11 have redundant functions in regulating skeletal development and patterning but not skeletal muscle size. We certainly cannot rule out the possibility that sufficient Gdf11 function is maintained by the small amount of *Gdf11 *expression remaining in the mutant muscles or that Gdf11 may have a function in developing skeletal muscle at early stages of development prior to activation of the *MLC *promoter. Gdf11 is detectable in serum [31] so we also cannot rule out the possibility that Gdf11 produced by other tissues not affected by muscle-specific recombination contributes to muscle mass regulation.

Nevertheless, the lack of an effect of these genetic manipulations of Gdf11 signaling on muscle mass was somewhat unexpected because myostatin and Gdf11 are highly homologous and because the function of myostatin in regulating skeletal muscle mass is clearly redundant with that of other TGFβ family members. Injection of a soluble Acvr2b receptor causes a 20% increase in muscle mass in *Mstn*^-/- ^mice demonstrating that at least one other ligand in addition to myostatin is a negative regulator of skeletal muscle size [15]. This other Acvr2b-binding ligand(s) must also be inhibited by follistatin; *Mstn*^-/- ^mice have double the muscle mass of *Mstn*^+/+ ^mice while *Mstn*^-/- ^mice overexpressing a muscle-specific *follistatin *transgene have quadruple the skeletal muscle mass of *Mstn*^+/+ ^mice [32]. Several other TGFβ superfamily members are potential candidates for myostatin functional redundancy. In addition to Gdf11, other family members, such as the activins and some bone morphogenetic proteins, bind Acvr2b and follistatin [31,33-36]. Myostatin, Gdf11, activin, and some BMPs can be isolated from serum using Acvr2b affinity purification [31]. The affinity of the BMPs for follistatin, however, is considerably lower than that of activin, myostatin, and Gdf11 [8,9]. Additionally, the activins inhibit differentiation of C2C12 myoblasts and skeletal muscle precursors in the chick limb and would therefore be likely candidates for functional redundancy with myostatin [31,37]. It is also possible there are multiple TGFβ family members that are negative regulators of muscle mass in addition to myostatin that individually have only small effects on muscle growth. If so, deletion or inhibition of these factors might cause a measurable increase in muscle mass only in the absence of myostatin and other redundant ligands.

Not only are the ligands redundant, myostatin and Gdf11 receptor function is also redundant. Although the affinity of myostatin for activin type II receptor (Acvr2, also known as ActRIIA) is low [2,5], *Acvr2*^-/- ^mice, like *Acvr2b*^-/- ^mice, have a small increase in skeletal muscle mass relative to *Mstn*^-/- ^mice [15]. This suggests that both Acvr2 and Acvr2b are receptors for myostatin in vivo. Similarly, *Acvr2b*^-/- ^mice have anterior homeotic transformations of the axial skeleton that are milder than those seen in *Gdf11*^-/- ^mice [38]. The *Acvr2*^+/- ^*Acvr2b*^-/- ^axial skeletal pattern closely phenocopies that of *Gdf11*^-/- ^mice [3] suggesting that both Acvr2 and Acvr2b are receptors for Gdf11. These data suggest therapeutic strategies that target both Acvr2 and Acvr2b receptor function or myostatin and other TGFβ growth factors with similar functions would lead to greater increases in muscle mass than targeting either Acvr2b or myostatin alone.

## Conclusion

Our results demonstrate that myostatin and Gdf11 have redundant functions in regulating skeletal patterning in mice. We did not, however, find evidence that Gdf11 is the TGFβ family member that is redundant to myostatin in regulation of skeletal muscle size. To determine the therapeutic strategy that results in the greatest potential increase in muscle mass, it will be important to identify the other TGFβ family members that negatively regulate skeletal muscle mass.

## Methods

### RNA isolation, Northern analysis

Total RNA was isolated from 8 week old animals from a pool of pectoralis, quadriceps, triceps, gastrocnemius, plantaris, and tibialis anterior muscles using Trizol reagent (Invitrogen). PolyA+ RNA was isolated from total RNA using an Oligotex mRNA kit (Qiagen). For Northern blot analysis, 5 μg mRNA was electrophoresed, blotted to GeneScreen Plus (Perkin Elmer), and hybridized according to the manufacturer's instructions using a probe corresponding to the carboxy-terminal region of *Gdf11 *in exon 3. After the signal decayed, the same blot was probed with *Gapdh *as a loading control.

### Muscle-specific targeting of *Gdf11*

All animal experiments were approved by the Institutional Animal Care and Use Committees of the Johns Hopkins University School of Medicine or NIDDK. To make muscle-specific *Cre *expressing mice, the coding sequence of *Cre *recombinase modified to contain Kozak consensus sites was inserted into the MDAF2 vector containing the myosin light chain promoter and 1/3 enhancer and SV40 processing sites [39]. To make the *Gdf11 *conditional allele, 3 *lox*P sites were inserted in intron 1 and flanking a *neo *gene downstream of the 3' UTR (Figure 2A) and used to replace the endogenous locus by gene targeting in ES cells. Microinjections and blastocyst injections were carried out by the Johns Hopkins School of Medicine Transgenic Core Facility. Offspring of male *Gdf11*^*flox*-*neo*/+ ^chimeras mated to C57BL/6 females were crossed to *EIIa-Cre *transgenic mice in the FVB background (The Jackson Laboratory) to generate males mosaic for *lox*P recombinations [40]. These males were mated to C57BL/6 females and screened by PCR for loss of the *neo *gene and retention of *Gdf11 *exons 2 and 3 (*Gdf11*^*flox*/+^). *Gdf11*^*flox*/+ ^mice were crossed once more to C57BL/6 mice, and *EIIA-Cre *negative *Gdf11*^*flox*/+ ^mice were crossed to *MLC-Cre *mice to generate *Gdf11*^*flox*/+ ^*MLC-Cre *mice. *Gdf11*^*flox*/+ ^*MLC-Cre *mice were mated to *Gdf11*^+/- ^in a 129/Sv genetic background from the original knockout line and to *Mstn*^+/- ^in the C57BL/6 genetic background. *Gdf11*^*flox*/+^*Mstn*^+/- ^*MLC-Cre *mice were crossed to *Gdf11*^*flox*/+^*Mstn*^+/- ^mice to generate *Mstn*^-/- ^*Gdf11*^*flox*/*flox *^and *Mstn*^-/- ^*Gdf11*^*flox*/*flox *^*MLC-Cre *mice which were interbred to produce animals for analysis. To show that *Gdf11*^Δ2–3 ^is a null allele, *Gdf11*^Δ2–3/+ ^mice with germline recombination of the *Gdf11*^*flox *^allele were obtained using *Cre/Esr1 *mice [41] purchased from The Jackson Laboratory.

### Genotyping

ES cell targeting and F1 offspring were analyzed by Southern blot after *Eco*RI digestion of genomic DNAs using the same probe as in ref. 18. *Eco*RI fragment sizes were 6.5 kb for *wild-type *and 2.5 kb targeted *Gdf11 flox-neo *alleles. Loss of the *neo *cassette after crosses to *EIIA-Cre *mice was determined by PCR using primers b and c. *Gdf11*^*flox*^*, Gdf11*^Δ2–3^, and *Gdf11*^+ ^alleles were detected by PCR using one reaction containing primers a, b, and c. *Cre *lines and *Mstn *alleles in crosses between *Mstn*^+/- ^and *Gdf11*^*flox*/+ ^mice were genotyped by PCR. All PCR was carried out at an annealing temperature of 55°C in buffer containing 10 mM Tris, pH 8.8, 25 mM KCl, 1.5 mM MgCl_2_, 0.2 mM of each dNTP, and 0.8 μM of each oligonucleotide primer. Oligonucleotide primers used and product sizes: *Cre*, 505 bp, 5' CCGAAATTGCCAGGATCAGGG 3' (forward) and 5' TCGCCATCTTCCAGCAGGCGC 3' (reverse); *Mstn*^+^, 220 bp, 5'-AGAAGTCAAGGTGACAGACACAC-3' (forward) and 5'-GGTGCACAAGATGAGTATGCGG-3' (reverse); *Mstn *null (in *PGK neo *cassette), 332 bp, 5'-GGATCGGCCATTGAACAAGATG-3' (forward) and 5'-GAGCAAGGTGAGATGACAGGAG-3' (reverse); *Gdf11 *alleles, *Gdf11*^*flox *^a/c = 300 bp, *Gdf11*^+ ^b/c = 359 bp, *Gdf11*^Δ2–3 ^b/c = 393 bp, (a) 5'ATGCAGATGGTAATACTTGGG3', (b) 5'-AAGGCTTGGGAAGCAGGCAAG-3', and (c) 5'-AGGTATGGTTAGGGTGTGGAG-3'. *Gdf11 *alleles were genotyped by Southern blot for crosses between the *Gdf11 *null allele from the original knockout line and *Gdf11*^*flox*/+ ^*MLC-Cre*. Genotyping of offspring from *Mstn*^+/- ^*Gdf11*^+/- ^crosses were performed by Southern blot as described [10,18].

### Analysis of muscles and skeletons

Soleus and EDL muscles from 8 week old (*Gdf11*^*flox*/-^, *Gdf11*^*flox*/- ^*MLC-Cre*, *Gdf11*^*flox*/*flox*^, and *Gdf11*^*flox*/*flox *^*MLC-Cre*) or 13 week old (*Mstn*^+/+ ^and *Mstn*^-/-^) mice were frozen and 12 μm cryostat sections were taken as described [10]. Fibers were counted from the widest part of the muscle belly from H/E (soleus) or trichrome (EDL) stained sections. Fiber typing was done on adjacent sections. Primary antibodies were used as dilutions of supernatants from hybridoma cell lines (American Type Culture Collection) and detected with the M.O.M. Peroxidase Kit (Vector Laboratories). Cell lines, dilutions, and fiber types detected were as follows: BA-D5, 1:30, type I; SC-71, 1:5, type IIA; BF-F3, 1:1, type IIB; N2.261, 1:5 plus BF-F3, 1:1, unstained type IID/X. Skeletons of newborn mice were stained as described [18].

### Statistical analysis

Body weight and muscle weights for the eight different genotypes produced from crosses between *Gdf11*^*flox*/+ ^*MLC-Cre *and *Gdf11*^+/- ^mice were analyzed by single-factor ANOVA with genotype as the factor and were considered significant if *P *< 0.05. All other comparisons between two genotypes were analyzed by Student's *t*-test.

## Abbreviations

Acvr2: activin type II receptor; Acvr2b: activin type IIB receptor; Gdf11: growth/differentiation factor 11; MLC: myosin light chain; Mstn: myostatin; TGFβ: transforming growth factor β.

## Authors' contributions

S-JL designed and produced the *Gdf11 *targeting construct. TVH constructed the *MLC-Cre *transgene and characterized the transgenic lines. ACM carried out ES cell targeting, performed all other experiments, and wrote the manuscript. All authors read and approved the final manuscript.

## Authors' information

Under a licensing agreement between MetaMorphix, Inc. (MMI) and the Johns Hopkins University, the authors are entitled to a share of royalty received by the University on sales of the factors described in this paper. We and the University own MMI stock, which is subject to certain restrictions under University policy. S-JL, who is the scientific founder of MMI, is a paid consultant to MMI and to Merck on research areas related to the study described in this paper. The terms of these arrangements are being managed by the University in accordance with its conflict of interest policies.

## Supplementary Material

Additional file 1 **Cranial and forelimb digit skeletal defects in *Mstn*^-/- ^*Gdf11*^-/- ^newborn mice. **(A and B) Skull phenotype of *Gdf11*^-/- ^(*A*) and *Mstn*^-/- ^*Gdf11*^-/- ^(*B*) pups. Double mutants have a rounded frontal bone (arrow). Forelimb digit phenotype of *Gdf11*^-/- ^(*C*) and *Mstn*^-/- ^*Gdf11*^-/- ^(*D*) pups. Digit identity is labeled with roman numerals. Note the fusion of digits III and IV and the supernumerary digit V (V*) in the double mutant. Click here for file
