# Supplementary material for: Redundancy of myostatin and growth/differentiation factor 11 function
Source: BMC Dev Biol. 2009 Mar 19;9:24. doi: 10.1186/1471-213X-9-24 (PMC2666675; doi:10.1186/1471-213X-9-24)
Supplement: Additional file 1 — Cranial and forelimb digit skeletal defects in Mstn-/- Gdf11-/- newborn mice. (A and B) Skull phenotype of Gdf11-/- (A) and Mstn-/- Gdf11-/- (B) pups. Double mutants have a rounded frontal bone (arrow). Forelimb digit phenotype of Gdf11-/- (C) and Mstn-/- Gdf11-/- (D) pups. Digit identity is labeled with roman numerals. Note the fusion of digits III and IV and the supernumerary digit V (V*) in the double mutant. [file 1471-213X-9-24-S1.pdf]

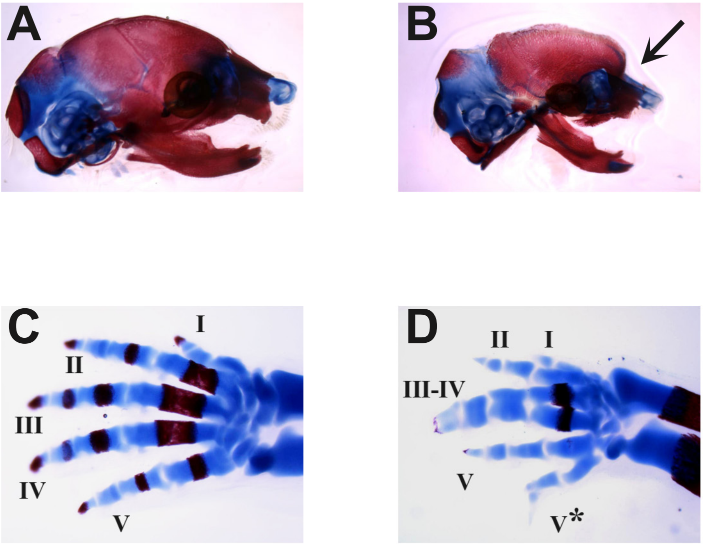

Additional File 1. Cranial and forelimb digit skeletal defects in *Mstn$^{-/-}$ Gdf11$^{-/-}$* newborn mice.
(A and B) Skull phenotype of *Gdf11$^{-/-}$* (A) and *Mstn$^{-/-}$ Gdf11$^{-/-}$* (B) pups. Double mutants have a rounded frontal bone (arrow). Forelimb digit phenotype of *Gdf11$^{-/-}$* (C) and *Mstn$^{-/-}$ Gdf11$^{-/-}$* (D) pups. Digit identity is labeled with roman numerals. Note the fusion of digits III and IV and the supernumerary digit V (V*) in the double mutant.
